# Getting antimalarials on target: impact of national roll-out of malaria rapid diagnostic tests on health facility treatment in three regions of Tanzania

**DOI:** 10.1111/tmi.12168

**Published:** 2013-08-13

**Authors:** Katia Bruxvoort, Admirabilis Kalolella, Happy Nchimbi, Charles Festo, Mark Taylor, Rebecca Thomson, Matthew Cairns, Julie Thwing, Immo Kleinschmidt, Catherine Goodman, S Patrick Kachur

**Affiliations:** 1 London School of Hygiene and Tropical MedicineLondon, UK; 2 Ifakara Health InstituteDar es Salaam, Tanzania; 3 Malaria Branch, Centers for Disease Control and PreventionAtlanta, GA, USA

**Keywords:** malaria, malaria rapid diagnostic test, implementation, artemisinin-based combination therapie, Tanzania, drug stock-outs, antibiotic use

## Abstract

**Objectives:**

Parasitological confirmation of malaria prior to treatment is recommended for patients of all ages, with malaria rapid diagnostic tests (mRDTs) an important tool to target artemisinin-based combination therapies (ACTs) to patients with malaria. To evaluate the impact on case management practices of routine government implementation of mRDTs, we conducted large-scale health facility surveys in three regions of Tanzania before and after mRDT roll-out.

**Methods:**

Febrile patients at randomly selected health facilities were interviewed about care received at the facility, and blood samples were collected for reference blood smears. Health facility staff were interviewed about their qualifications and availability of malaria diagnostics and drugs.

**Results:**

The percentage of febrile patients tested for malaria at the facility increased from 15.8% in 2010 to 54.9% in 2012. ACTs were obtained by 65.8% of patients positive by reference blood smear in 2010 and by 50.2% in 2012 (*P* = 0.0675); no antimalarial was obtained by 57.8% of malaria-negative patients in 2010 and by 82.3% in 2012 (*P* < 0.0001). Overall, ACT use decreased (39.9–21.3%, *P* < 0.0001) and antibiotic use increased (31.2–48.5%, *P* < 0.0001).

**Conclusion:**

Roll-out of mRDTs in Tanzania dramatically improved diagnostic testing for malaria and reduced overuse of ACTs for patients without parasitemia. However, post–roll-out almost 50% of febrile patients did not receive a diagnostic test, and almost 50% of patients testing positive did not receive ACTs. Stock-outs of ACTs and mRDTs were important problems. Further investigation is needed to determine reasons for not providing ACTs to patients with malaria and potential for inappropriate antibiotic use.

## Introduction

Artemisinin-based combination therapies (ACTs) are the first-line drugs for malaria in most endemic countries, but there are concerns that targeting of ACTs to those in need remains poor. Many patients with malaria do not obtain ACTs, while many others with febrile illness obtain ACTs but do not have malaria parasitaemia (Whitty *et al.* 2008). Because microscopic testing of malaria has been limited in availability and is often of poor quality (McMorrow *et al.* 2008; Drakeley & Reyburn 2009; Kahama-Maro *et al.* 2011), it has been standard practice to diagnose malaria presumptively based on fever, resulting in over-treatment with antimalarials and under-treatment of non-malarial infections (Reyburn *et al.* 2004; D'Acremont *et al.* 2009). Such non-malarial febrile illnesses in a minority of cases are caused by bacterial infections but are most often due to self-limiting viral illnesses (Mtove *et al.* 2011; Leslie *et al.* 2012; McMorrow *et al.* 2012). Malaria rapid diagnostic tests (mRDTs) are thought to be an important tool to improve malaria diagnosis and targeting of ACTs (Murray *et al.* 2008; Drakeley & Reyburn 2009). Parasitological confirmation of malaria prior to treatment is now recommended for patients of all ages by World Health Organization (2010), with mRDTs an important part of the ‘T3: Test, Treat, Track’ initiative to ensure that every suspected malaria case is tested and every confirmed case is treated and tracked in a surveillance system (World Health Organization 2012). Decreases in the proportion of febrile illnesses associated with malaria in many settings (D'Acremont *et al.* 2010) have led commentators to stress the urgency of expanding mRDT coverage (D'Acremont *et al.* 2009), although important challenges to effective implementation have been highlighted (McMorrow *et al.* 2008; English *et al.* 2009).

Malaria rapid diagnostic test implementation has had a varied impact on clinical decisions. While some studies have demonstrated significant reductions in the proportion of patients obtaining an antimalarial drug (Williams *et al.* 2008; Msellem *et al.* 2009; Thiam *et al.* 2011; Yukich *et al.* 2012) or reductions in over-treatment of malaria (Kyabayinze *et al.* 2010; Bastiaens *et al.* 2011; D'Acremont *et al.* 2011; Masanja *et al.* 2012), others have reported under-utilisation of diagnostic tests (Hamer *et al.* 2007; Nyandigisi *et al.* 2011) and frequent antimalarial treatment of patients with negative test results (Hamer *et al.* 2007; Reyburn *et al.* 2007; Bisoffi *et al.* 2009; Skarbinski *et al.* 2009; Nyandigisi *et al.* 2011). This variability has been documented across studies within the same country. For example, in Tanzania, D'Acremont *et al*. (2011) demonstrated a reduction in over-treatment with antimalarials, while Reyburn *et al*. (2007) did not, and McMorrow *et al*. (2008) reported significant challenges in both microscopy and mRDT use under routine conditions. Another key concern is that increased mRDT use will lead to greater and often inappropriate use of antibiotics as clinicians are faced with unclear treatment decisions for mRDT-negative patients, with potentially adverse consequences for antibiotic resistance (Baiden *et al.* 2012).

Artemether–lumefantrine (ALu) has been the first-line drug for treatment of malaria in Tanzania since 2004, although roll-out to the public sector did not begin until 2006. Treatment at government hospitals, health centres and dispensaries is intended to be free of charge for children below 5 years old and pregnant women, although this policy is not always adhered to (Njau *et al.* 2006). In 2006, the National Malaria Control Programme revised policies favouring presumptive diagnosis of malaria to treatment based on parasitological confirmation for patients aged 5 years and above, with treatment based on clinical diagnosis permitted for children under age 5 years (United Republic of Tanzania Ministry of Health & Social Welfare 2006), although implementation remained limited in practice. In 2010, the policy was amended to include parasitological confirmation of suspected malaria cases for all ages (United Republic of Tanzania Ministry of Health & Social Welfare 2011), corresponding with the phased roll-out of mRDTs to all levels of government health facilities from 2009 to 2012.

While mRDT implementation has been studied in many settings, very few studies have reported evaluations of mRDT roll-out under routine operational conditions, where implementation has been purely the responsibility of the government (Bastiaens *et al.* 2011; Thiam *et al.* 2011; Masanja *et al.* 2012; Yukich *et al.* 2012). Two studies from Senegal and Zambia used routinely collected data to measure the impact on antimalarial consumption but did not assess the appropriateness of case management (Thiam *et al.* 2011; Yukich *et al.* 2012), while two studies in Tanzania examined very early results of mRDT roll-out in limited settings [two hospitals (Bastiaens *et al.* 2011) and one demographic surveillance site (Masanja *et al.* 2012)]. More commonly, study teams in these studies had participated in implementation to some degree, potentially influencing the findings and limiting their generalisability.

In this article, we report the impact on case management practices of routine government implementation of provision and use of mRDTs in Tanzania, based on large-scale health facility surveys conducted before and after mRDT roll-out. The surveys were conducted in three of mainland Tanzania's 21 regions, reflecting the country's diversity of malaria epidemiology and commodity availability, thereby allowing us to explore the impact of policy implementation under a range of contexts.

## Methods

### Context

This study was part of the IMPACT2 project, which took place in Mwanza, Mbeya and Mtwara regions of Tanzania, with populations of approximately 3 771 000, 2 822 000 and 1 375 000, respectively (National Bureau of Statistics (Tanzania) 2006). Each region includes a large municipality and smaller district towns, although the populations are predominantly rural. A greater percentage of the population is in the poorest national wealth quintile in Mtwara (35.5%), compared with Mwanza (20.8%) and Mbeya (7.7%) (National Bureau of Statistics (Tanzania) & ICF Macro 2011). According to the 2011–2012 HIV and Malaria Indicator Survey, Mwanza and Mtwara have moderately high malaria prevalence (18.6% and 17.4% among children 6–59 months of age), while Mbeya has low prevalence (0.5%) (Tanzania Commission for AIDS (TACAIDS) 2012). Malaria treatment in the public sector is provided by regional and district hospitals and a network of health centres and smaller dispensaries.

Roll-out of mRDTs took place in February 2011 in Mwanza and Mbeya, but not until May 2012 in Mtwara. A limited number of facilities in Mwanza and Mtwara had participated in small-scale mRDT pilots prior to roll-out. Roll-out involved training for 1 week of regional representatives and four representatives from each district, who then in turn trained one or two health workers from each public facility, usually the incharge clinician and laboratory technician or another health worker. The mRDT-specific training built upon earlier trainings on case management of malaria with ACTs, some of which had previously included instruction on performing mRDTs. Content included the rationale for using mRDTs, the mRDT algorithm for case management, reporting procedures for monitoring of mRDT use and the importance of compliance to test results. The trainings included demonstrations and practical exercises on performing mRDTs and reading results. Health workers receiving formal training were expected to pass on the information to colleagues who had not attended in a cascade-training approach. An initial stock of mRDTs was supplied to each facility, after which additional mRDTs could be ordered based on reported consumption.

### Evaluation design and sample size

**Figure 1:**
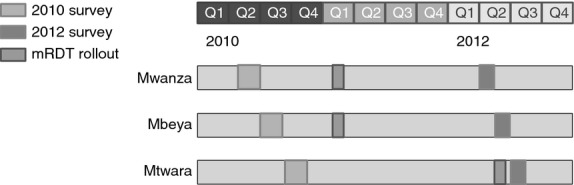
Timing of surveys and malaria rapid diagnostic test rollout.

Baseline health facility surveys took place in 2010, during May–June in Mwanza, July–August in Mbeya and September–October in Mtwara. Post-implementation surveys followed in 2012 in Mwanza in April–May, Mbeya in May–June and Mtwara in June–July (Figure1).

The sample size for the study was calculated to detect a 15% absolute change in a composite indicator of appropriate treatment given true malaria parasitaemia status, as identified by reference blood smears collected by study staff. The composite indicator was defined as the proportion of patients with fever or history of fever who had a malaria-positive reference blood smear and received ACT at the health facility, or had a malaria-negative reference blood smear and did not receive any antimalarial. Assuming 5% significance, 80% power, a baseline proportion receiving appropriate treatment of 65%, 5% refusal and a design effect of 2.5, 338 patients with fever or history of fever per study region were required in each survey round.

### Data collection

In each region, 35 health facilities in 2010 and 60 in 2012 were randomly selected with probability proportional to malaria outpatient utilisation, based on the most recent data available for all government health facilities. Due to slower than expected recruitment in 2010, additional facilities were added from a list of randomly selected alternates for each region, and the total number of facilities selected in 2012 was increased. A similar number of patients were sampled at each facility, resulting in an approximately self-weighting survey sample. Each facility was visited by the study team for 1 day. Patients presenting for outpatient care on the day of the study team's visit with fever or history of fever in the previous 48 hours were enrolled upon arrival at the health facility, subject to informed consent having been obtained. To achieve sufficient sample size, facilities were replaced with randomly selected alternate facilities of similar size if no eligible patients attended the facility on the day of the study team's visit. In 2010, the first 12 eligible patients under five and the first 12 eligible patients aged five and above were enrolled. Because this target was not met at most facilities in 2010 and additional randomly selected facilities had to be added, the protocol was adjusted in 2012 to enrol all patients meeting eligibility criteria at each facility during daytime operating hours.

After completion of their consultations with facility staff, patients were interviewed by research staff about demographic information, previous treatment for fever and care received at the facility, including blood tests for malaria conducted as part of the consultation (facility tests) and drugs obtained. Health facility staff seeing enrolled patients were interviewed about their qualifications, training, knowledge, and availability of antimalarial drugs, mRDTs, microscopy and antibiotics.

### Definitions

Facilities were considered to have mRDTs in stock if the study team observed at least one full box containing 25 non-expired mRDTs, and facilities were considered to have microscopy upon observation of a functional microscope, slides and Giemsa stain for at least 25 smears, and a microscopist. Facilities were considered to have ALu in stock if they had had at least one observed, non-expired blister pack for any age/weight group. Antibiotics were considered to be in stock if any non-expired oral antibiotics were observed.

Finger prick blood samples were taken from enrolled patients by study staff to test for malaria parasitaemia by study mRDTs (ICT Diagnostics, Cape Town, South Africa) and thick blood smears (reference blood smears) stained with 10% Giemsa. Reference blood smears were double-read at the Ifakara Health Institute in Bagamoyo by two microscopists blinded to the initial reading and patients' study mRDT results, with discrepant readings read by a third microscopist. Parasites were counted against 200 white blood cells, and slides were considered negative if no parasites were found after examining 100 fields.

### Ethics

All questionnaires and consent forms were translated into Swahili and piloted by native speakers. The study protocol was approved by the ethical review boards of the Ifakara Health Institute and the London School of Hygiene and Tropical Medicine. CDC investigators provided technical assistance in design and analysis but were not engaged in data collection. Written informed consent was obtained from health workers and patients or their caregivers prior to enrolment. Patients who tested positive by the study mRDT and had not already received ACT were given the weight-appropriate blister pack by study staff. Pregnant women in the first trimester and children below 5 kg who tested positive were referred back to the health facility incharge for treatment decisions.

### Statistical analysis

Data were collected using personal digital assistants (PDAs) and were analysed in Stata 11.0 (Stata Corporation, College Station, USA) using survey commands to account for the two-stage survey design with stratification by region. Percentages with 95% confidence intervals and p-values for the Pearson design-based *F*-test were reported where applicable, with significance defined as *P *< 0.05.

## Results

We visited 140 health facilities (11 hospitals, 24 health centres and 105 dispensaries) between May and October 2010, and 176 health facilities (13 hospitals, 31 health centres, 132 dispensaries) between April and July 2012.

### Availability of diagnostics and drugs

**Figure 2:**
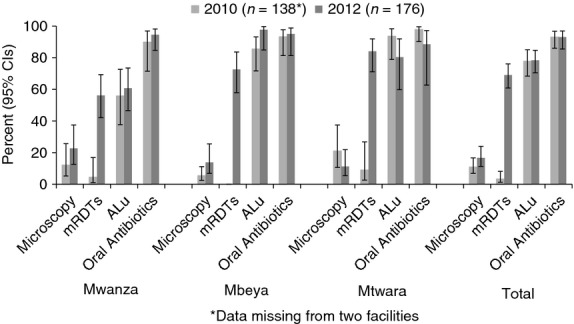
Availability of malaria diagnostics, artemether–lumefantrine and oral antibiotics in health facilities in 2010 (pre-roll-out) and 2012 (post-roll-out).

In 2010, only 3.3% of health facilities had mRDTs in stock, while 10.9% had microscopy and 11.8% had either mRDTs or microscopy available (Figure2). In 2012, mRDTs were in stock in 69.0% of health facilities, with microscopy available at 16.7% and either diagnostic test available at 73.9%. The percentage of facilities with any ALu in stock was similar in both years at 77.8% in 2010 and 78.4% in 2012. All four age/weight blister packs were in stock in only 41.1% of health facilities in 2010 and 27.3% in 2012. Stock-outs of all four packs at the same time in the previous 3 months had been experienced by 35.5% of facilities in 2010 and 41.6% in 2012. During both surveys, stock-outs of ALu were most severe in Mwanza, which also faced the most mRDT stock-outs in 2012. At least one oral antibiotic (mainly cotrimoxazole and amoxicillin) was in stock at 93.1% of health facilities in both 2010 and 2012, with little difference by region.

### Health worker characteristics and training

We interviewed 154 health workers in 2010 and 209 in 2012. While most health worker characteristics remained similar between 2010 and 2012 (Table1), the percentage of health workers who had attended a formal in-service training on mRDTs (including training on ACTs where health workers were taught how to use mRDTs) increased from 6.5% in 2010 to 59.4% in 2012. Mtwara, the most recent region to receive mRDT roll-out, had the highest percentage of mRDT-trained health workers (79.0%), compared with 49.3% in Mwanza and 58.8% in Mbeya.

**Table 1:** Health worker characteristics (Percent, 95% Confidence interval)

Health workers with interview data (N)	Mwanza		Mbeya		Mtwara		Total	
	2010	2012	2010	2012	2010	2012	2010	2012
	41	71	64	72	49	66	154	209
Age (years)								
25-34	15.1 (5.2, 36.9)	34.3 (23.7, 46.8)	29.2 (14.7, 49.7)	31.7 (19.9, 46.3)	10.5 (4.2, 23.7)	21.4 (10.9, 37.7)	20.7 (12.6, 32.0)	30.6 (23.6, 38.7)
35-44	32.5 (17.9, 51.6)	15.7 (8.4, 27.4)	19.8 (9.6, 36.5)	9.2 (4.1, 19.5)	23.7 (11.4, 43.0)	19.6 (10.1, 34.6)	24.7 (16.6, 35.2)	14.5 (9.8, 20.9)
45 and older	52.4 (34.4, 69.8)	50.0 (37.4, 62.5)	51.0 (30.2, 71.3)	59.1 (45.0, 71.9)	65.8 (46.4, 81.1)	59.0 (42.2, 73.9)	54.7 (42.4, 66.4)	55.1 (47.4, 62.6)
Male sex	71.3 (51.9, 85.1)	65.8 (54.4, 75.6)	47.5 (27.6, 68.2)	49.3 (34.9, 63.8)	39.8 (23.3, 59.0)	43.2 (26.9, 61.0)	53.4 (40.8, 65.6)	52.9 (45.2, 60.4)
Qualifications								
Medical or Clinical Officer	55.8 (37.4, 72.8)	70.1 (55.9, 81.2)	41.9 (24.1, 62.1)	40.9 (27.5, 55.8)	54.9 (33.8, 74.4)	36.3 (22.9, 52.1)	49.1 (37.0, 61.4)	52.7 (43.3, 61.8)
Nurse, midwife or assistant clinical officer	39.3 (22.9, 58.5)	27.2 (16.8, 40.9)	28.7 (14.6, 48.4)	46.8 (32.5, 61.7)	36.5 (17.6, 60.7)	50.5 (34.0, 66.9)	33.7 (23.3, 46.1)	39.0 (30.4, 48.4)
Assistant or attendant	4.9 (1.0, 20.7)	2.7 (0.7, 9.9)	29.5 (10.6, 59.7)	12.3 (6.1, 23.0)	8.6 (3.0, 22.2)	13.2 (6.1, 26.3)	17.1 (7.1, 36.0)	8.3 (5.2, 13.1)
Number of patients seen on a regular day								
15 or less	27.0 (13.7, 46.2)	29.7 (17.9, 45.0)	70.9 (54.0, 83.5)	49.9 (35.5, 64.2)	31.3 (16.8, 50.6)	23.3 (12.8, 38.8)	48.3 (36.4, 60.5)	34.8 (26.6, 43.9)
16-30	50.5 (32.7, 68.1)	53.6 (40.2, 66.5)	25.3 (14.1, 41.2)	38.3 (25.6, 52.9)	63.2 (43.6, 79.2)	64.0 (47.8, 77.5)	41.6 (30.7, 53.3)	51.1 (42.7, 59.5)
More than 30	22.5 (11.5, 39.6)	16.7 (6.6, 36.2)	3.8 (1.6, 8.9)	11.8 (5.8, 22.6)	5.6 (1.5, 18.9)	12.7 (6.0, 24.8)	10.1 (6.0, 16.6)	14.1 (8.3, 23.2)
Attended formal training on ACTs in the last 5 years	70.3 (51.4, 84.2)	47.3 (34.7, 60.2)	40.2 (23.2, 60.0)	61.1 (47.1, 73.4)	77.1 (60.9, 88.0)	73.6 (55.1, 86.3)	57.8 (44.6, 70.0)	58.0 (49.5, 66.0)
Attended formal training on how to use mRDTs in the last 5 years*	8.1 (2.3, 25.9)	49.3 (33.6, 65.2)	3.8 (0.7, 18.4)	58.8 (44.5, 71.7)	9.8 (3.6, 24.4)	79.0 (60.0, 90.5)	6.5 (3.0, 13.5)	59.4 (49.4, 68.7)
Received supervision on ACTs in the last six months	31.8 (17.5, 50.6)	30.8 (20.0, 44.2)	19.1 (9.8, 33.9)	29.1 (18.0, 43.4)	48.2 (29.1, 67.9)	19.2 (10.1, 33.3)	29.5 (20.9, 39.8)	27.5 (20.7, 35.5)
Reported having ACT job aid in consultation room	78.4 (59.3, 90.1)	62.2 (49.3, 73.6)	64.1 (37.7, 84,1)	66.4 (52.5, 78.0)	88.6 (74.2, 95.4)	77.7 (63.3, 87.6)	74.0 (58.0, 85.4)	67.3 (59.5, 74.3)
Correctly identified correct dosage regimen of ACT for a 10 kg child	65.9 (47.4, 80.6)	92.7 (83.6, 96.9)	81.9 (66.4, 91.2)	92.0 (81.4, 96.8)	87.1 (71.2, 94.8)	90.9 (79.3, 96.3)	77.9 (68.3, 85.3)	92.0 (86.9, 95.2)
Correctly identified correct dosage regimen of ACT for an adult	96.0 (75.7, 99.5)	98.5 (89.5, 99.8)	100.0	98.4 (89.4, 99.8)	100.0	90.0 (63.6, 97.9)	98.7 (91.2, 99.8)	96.4 (89.2, 98.9)

* Includes formal training on ACTs where health workers were taught how to use mRDTs.

### Patient characteristics

Of 1746 patients interviewed in 2010, 59.2% were under 5 years old, while in 2012, 66.2% of 1710 patients were under-fives (Table2). A lower percentage of patients had sought care at another source prior to coming to the study health facility in 2010 compared with 2012 (18.8% versus 32.4%, *P* < 0.0001). There was no evidence of change from 2010 to 2012 in the percentage testing positive by study mRDT in any of the three regions or overall (20.8% and 22.9%, respectively, *P* = 0.4), but the percentage of children over age five and adults testing positive by study mRDT increased from 17.0% in 2010 to 24.3% in 2012 (*P* = 0.0116). In Mwanza and Mbeya, there was no evidence of change in the percentage with a positive reference blood smear, but in Mtwara, there was an increase from 20.9% in 2010 to 31.8% in 2012 (*P* = 0.0048), primarily reflecting the significant increase in parasitaemia among older children and adults.

**Table 2:** Patient characteristics (Percent, 95% Confidence interval)

	Mwanza		Mbeya		Mtwara		Total	
	2010	2012	2010	2012	2010	2012	2010	2012
Patients with interview data (*N*)	689	750	559	388	498	572	1746	1710
Percent below 5 years	58.8 (51.4, 65.8)	68.5 (63.0, 73.6)	55.1 (48.1, 61.9)	63.7 (58.0, 69.0)	64.3 (58.5, 69.6)	64.9 (59.7, 69.7)	59.2 (55.1, 63.1)	66.2 (63.0, 69.2)
Male sex	43.4 (38.8, 48.1)	43.6 (40.2,47.1)	42.6 (38.0, 47.3)	43.0 (38.6, 47.6)	44.0 (40.4, 47.6)	41.6 (37.6, 45.7)	43.3 (40.8, 45.9)	42.8 (40.6, 45.1)
Sought care at another source before coming to health facility	30.8 (26.7, 35.2)	38.4 (34.5, 42.5)	10.6 (7.4, 14.9)	30.2 (24.4, 36.6)	11.5 (7.2, 17.7)	25.9 (20.6, 31.9)	18.8 (16.3, 21.5)	32.4 (29.5, 35.4)
Can reach health facility within one hour	69.1 (63.8, 74.0)	72.4 (67.4, 76.9)	74.8 (69.3, 79.6)	69.7 (62.6, 76.0)	68.5 (61.1, 75.0)	65.1 (59.8, 70.1)	70.8 (67.4, 74.0)	69.3 (66.1, 72.3)
Slept under insecticide-treated net* last night	81.7 (77.5, 85.2)	88.3 (85.1, 91.0)	75.8 (71.7, 79.5)	79.5 (74.8, 83.5)	81.9 (77.4, 85.7)	84.5 (80.8, 87.6)	80.0 (77.5, 82.1)	85.1 (83.0, 87.0)
Median days since fever onset	3	2	2	2	2	2	2	2
Percent testing positive by study mRDT†	21.3 (16.6, 26.9)	14.4 (9.8, 20.8)	5.9 (3.5, 9.9)	6.5 (3.3, 12.1)	37.0 (29.8, 44.7)	45.1 (38.6, 51.8)	20.8 (17.7, 24.4)	22.9 (19.5, 26.7)
Percent testing positive by study blood smear‡	6.6 (4.4, 9.8)	10.7 (7.1, 15.7)	1.6 (0.7, 3.5)	4.8 (2.2, 10.0)	20.9 (16.4, 26.3)	31.8 (27.1, 36.8)	9.2 (7.4, 11.4)	16.5 (13.9, 19.4)

* A long-lasting insecticide-treated net (LLIN) obtained within the previous 3 years or a conventional net treated with insecticide in the previous year.

† *N* = 1737 in 2010 and 1707 in 2012 due to 12 interviewed patients without study RDT results.

‡ *N* = 1649 in 2010 and 1687 in 2012 due to 120 interviewed patients with missing blood smears.

### Use of malaria diagnostics

**Figure 3:**
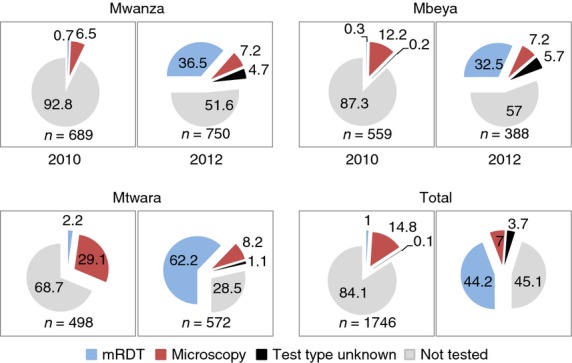
Percent of patients receiving malaria diagnostic test at health facility pre and post malaria rapid diagnostic test roll-out.

The results show a large increase in the use of malaria diagnostics post-implementation (Figure3). In 2010, 7.2% of febrile patients in Mwanza, 12.7% in Mbeya and 31.3% in Mtwara were tested for malaria by health workers at the facility, with almost all of these patients tested by microscopy. By 2012, this had increased to 48.4% in Mwanza, 43% in Mbeya and 71.5% in Mtwara, which had most recently received mRDT roll-out. If the analysis for 2012 is restricted to facilities with diagnostics in stock, the percentage tested increases to 67.4% in Mwanza, 49.0% in Mbeya and 82.1% in Mtwara. In both surveys, children under 5 years old were no more likely to be tested than older children and adults. The majority of malaria tests in 2012 were mRDTs (80.5%). Facility tests were positive for almost half of febrile patients in Mtwara (46.5%), compared with 22.8% in Mwanza and 11.4% in Mbeya. Overall, sensitivity and specificity of facility blood smears compared with reference blood smears in 2012 were 70.6% and 63.7%, respectively, with a positive predictive value of 24.5% and a negative predictive value of 92.9% (*n* = 119 patients). Sensitivity and specificity of facility mRDTs compared with reference blood smears were 91.3% and 88.0%, respectively, with a positive predictive value of 69.3% and a negative predictive value of 97.3% (*n* = 746).

### Treatment according to facility test results

We present results on treatment obtained firstly according to the facility test and secondly according to the reference blood smear. Results in relation to the facility test are presented for 2012 only, as few facility tests were conducted in 2010. As explained above, we define appropriate antimalarial treatment for patients with positive test results as obtaining an ACT and for those with negative results as not obtaining an antimalarial. Only three women in 2010 and three women in 2012 reported being in the first trimester of pregnancy and had a positive reference blood smear, and of these, only one in 2010 received any treatment (quinine). Due to their negligible numbers, these women were not excluded from the analysis.

**Figure 4:**
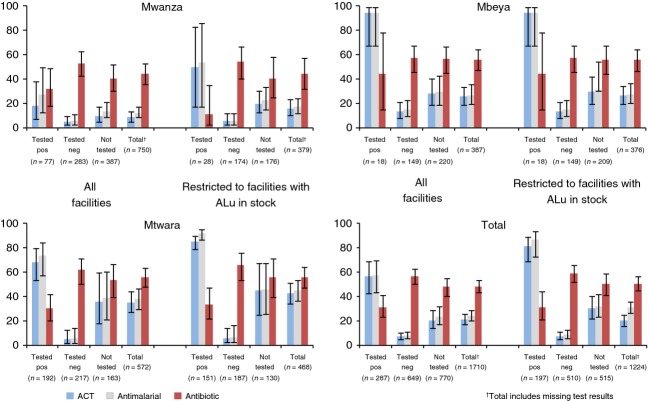
Treatment obtained at health facilities according to facility malaria test (microscopy or mRDT) post-roll-out (Percent, 95% CIs).

In 2012, 56.5% of patients testing positive by the facility test obtained an ACT, while 7.7% of those testing negative and 23.6% of those not tested obtained any antimalarial (Figure4). In comparison, 25.2% of those testing negative in 2010 obtained any antimalarial (denominator of *n* = 131), although very few patients overall were tested. While the percentage of patients in 2012 testing negative who obtained any antimalarial was low in all three regions, the percentage testing positive that obtained ACT varied from 18.2% in Mwanza to 68.2% in Mtwara and 94.6% in Mbeya. Older children and adults testing positive were more likely to obtain an ACT than children under five who tested positive (67.7% and 50.3%, respectively, *P* = 0.0026). Considering only facilities where ALu was in stock, 81.2% of patients testing positive by the facility test obtained an ACT, and of those who did not, 70.3% did not obtain any antimalarial, while 29.7% were treated with quinine. Of patients testing negative and of those not tested at facilities with ALu in stock, 8.6% and 32.0%, respectively, obtained any antimalarial. In both surveys, almost all antimalarials obtained were ACTs (92.2% in 2010 and 88.4% in 2012), and almost all ACTs were ALu. In 2012, antibiotics were obtained by 31.5% of those positive for malaria by the facility test, 56.9% of those testing negative and 47.9% of those not tested.

### Treatment according to reference blood smear results

**Figure 5:**
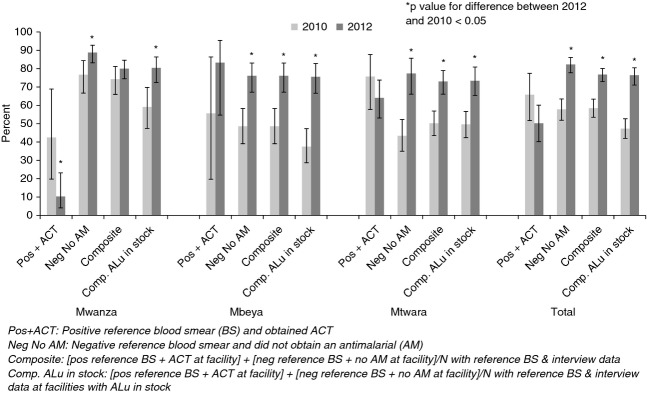
Patients receiving correct treatment according to parasitaemia status by study blood smear pre and post mRDT roll-out.

We assessed treatment obtained against reference blood smear results in 2010 and 2012 (Figure5). Based on reference blood smears, the composite indicator of appropriate treatment increased from 58.5% in 2010 to 76.8% (*P* < 0.0001). Mwanza appeared to perform very well by this composite indicator (80.0% in 2012), although to a large degree this reflected patients testing negative being unable to obtain ACTs at facilities where they were not in stock. To address this concern, the analysis was restricted to facilities where ALu was in stock, showing an overall improvement in the percentage of patients obtaining appropriate treatment according to the reference blood smear from 47.3% in 2010 to 76.4% in 2012 (*P* < 0.0001).

As reported above for the facility tests, we also report appropriate treatment for patients with positive reference blood smears and negative reference blood smears separately. ACTs were obtained by 65.8% of patients testing positive in 2010 (denominator of *n* = 152) and 50.2% in 2012 (*n* = 287) (*P *= 0.0675), while no antimalarial was obtained by 57.8% of patients testing negative in 2010 and 82.3% in 2012 (*P* < 0.0001). In 2012, but not 2010, older children and adults with a positive reference blood smear were more likely to obtain ACTs than children under five (45.0% and 60.0%, respectively, *P* = 0.0176). Considering only facilities where ALu was in stock, ACTs were obtained by 82.6% of patients of all ages testing positive in 2010 (*n* = 121) and 71.8% in 2012 (*n* = 188) (*P* = 0.0756), while no antimalarial was obtained by 43.2% of patients testing negative in 2010 and 77.3% in 2012 (*P* < 0.0001). In Mwanza, the percentages of patients positive by reference blood smear (*n* = 40 in 2010 and 78 in 2012) who obtained ACT were very low both years, especially in 2012 (42.5% and 10.3%, respectively), with little improvement observed when considering only facilities with ALu in stock (73.9% and 28.6%, respectively, *n* = 23 in 2010 and 21 in 2012) (*P *< 0.0161).

### Overall use of ACTs and antibiotics

**Figure 6:**
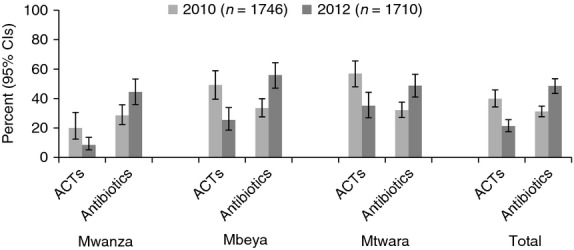
Patients receiving ACT and antibiotic by region pre and post mRDT roll-out.

The consequences for overall levels of ACT and antibiotic treatment are shown in Figure6. Overall, the percentages of patients obtaining ACT decreased significantly (39.9% to 21.3%, *P* < 0.0001) between 2010 and 2012, while the proportion obtaining an antibiotic increased significantly (31.2% to 48.5%, *P* < 0.0001). The same pattern occurs in all three regions and for both patients under and over 5 years of age. The percentage obtaining both ACT and antibiotics did not change overall between 2010 and 2012 (11.7% and 8.3%, *P* = 0.10). Compared to all patients in 2010, antibiotic use was higher in 2012 not only in the subgroup testing negative by the facility test, but also in the subgroup not tested (Figure4).

## Discussion

We have presented results from large-scale health facility surveys in three regions of Tanzania before and after the roll-out of mRDTs in all levels of government health facilities. We have reported on the availability and use of diagnostic testing among febrile patients and case management with ACTs based on facility test results and reference blood smears, and we have also examined the effect of this policy change on prescription of antibiotics. The findings are representative of Mwanza, Mbeya and Mtwara Regions, which encompass considerable diversity in malaria transmission, economic status and culture.

Malaria rapid diagnostic test roll-out in Tanzania led to substantial changes in the provision of malaria case management in public facilities. Post-roll-out nearly 70% of facilities had mRDTs in stock and 60% of health workers had received formal mRDT training. This was associated with a large increase in the proportion of febrile outpatients tested for malaria from 15.5% in 2010 to 54.9% in 2012, and facility mRDT testing was shown to have relatively good sensitivity and specificity. However, there were still major gaps in diagnostic coverage, with almost half of all patients not tested. In Mtwara, the proportion of patients tested (71.5%) and the proportion of health workers with formal mRDT training (79.0%) were higher than in Mwanza and Mbeya, reflecting the more recent roll-out, and therefore, lower potential for training coverage to be eroded by staff turnover or for mRDT stock-outs to arise. The latter was an important factor in suboptimal diagnostic coverage, with the per cent tested increasing to 70% if only facilities with diagnostics available were considered. Other studies have similarly reported less-than-optimal coverage of diagnostic tests (Hamer *et al.* 2007; Nyandigisi *et al.* 2011; Masanja *et al.* 2012). In addition to poor diagnostic availability (Ezeoke *et al.* 2012), reasons for poor coverage may include inadequate health worker training, negative health worker perceptions of mRDTs, provider workload or patient preferences (Chandler *et al.* 2008a; Williams *et al.* 2008; Asiimwe *et al.* 2012; Baiden *et al.* 2012). Health centre and dispensary patients paid a flat rate for treatment, so patient willingness-to-pay for tests is not expected to have been a factor in these facilities, but may have contributed in hospital settings where separate fees were sometimes charged for diagnostics.

Provider compliance with test results in terms of prescription has been a major concern in the roll-out of improved services for parasitological confirmation. Reported reasons for low compliance include providers' or patients' lack of confidence in the test result, the presence of symptoms associated with malaria, and the lack of alternative diagnoses identified by the providers or accepted by the patients ([Bibr b6][Bibr b7], 2010; Asiimwe *et al.* 2012; Ezeoke *et al.* 2012). While some studies have reported poor compliance with negative test results (Hamer *et al.* 2007; Reyburn *et al.* 2007; Bisoffi *et al.* 2009; Nyandigisi *et al.* 2011). our findings correspond with other studies that have demonstrated a substantial reduction in antimalarial treatment after implementation of mRDTs (Williams *et al.* 2008; Msellem *et al.* 2009; Kyabayinze *et al.* 2010; Bastiaens *et al.* 2011; D'Acremont *et al.* 2011; Thiam *et al.* 2011; Masanja *et al.* 2012; Yukich *et al.* 2012). Our results show that treatment of patients with a negative facility test in 2012 was less than one in ten, even in facilities with ALu in stock. The proportion of patients negative by reference blood smear who did not receive any antimalarial increased significantly from 57.8% in 2010 to 82.3% in 2012.

The reduction in ACT provision for patients who were not parasitemic led to a significant increase in the composite indicator of patients appropriately treated of 18 percentage points including all facilities and of 29 percentage points considering only those facilities with ALu in stock. However, while over-prescription of ACT was substantially reduced, under-prescription remained a major problem. Only 56.5% of patients with a positive facility test obtained an ACT in 2012, with particularly poor results in Mwanza, where only 18.2% of positive patients obtained ACT. There was weak evidence of a decrease in the proportion of patients positive by reference blood smear who obtained ACT between 2010 and 2012 overall, and a significant fall in Mwanza, although the percentages of patients testing positive overall were low (9.2% in 2010 and 16.5% in 2012) resulting in small subsamples and large confidence intervals. Poor ACT availability was a major factor in the under-treatment of patients testing positive. ACT stock-outs were present in all regions but especially severe in Mwanza, with around 40% of facilities experiencing complete ACT stock-outs at the time of both surveys. Even at facilities where ALu was in stock, nearly 20% of patients overall with a positive facility test result did not obtain ACTs. Fear of future stock-outs prompting drug rationing or the absence of the appropriate weight-specific blister packs may have discouraged health workers from dispensing ACT to some patients testing positive by the facility test (Wasunna *et al.* 2008). Again at health centres and dispensaries, this is unlikely to be due to patients not wanting to pay for ACTs as flat fees for consultations were generally charged, but willingness-to-pay could have been a factor in hospitals which sometimes charged separately for drugs. These results are in contrast to other studies that found more than 90% compliance with positive test results (Hamer *et al.* 2007; McMorrow *et al.* 2008; Williams *et al.* 2008; Bisoffi *et al.* 2009; Msellem *et al.* 2009; Kyabayinze *et al.* 2010; D'Acremont *et al.* 2011), perhaps reflecting the greater risk of ACT stock-outs and more limited health worker training coverage and supervision under the routine, operational conditions we evaluated. Another study evaluating early effects of mRDT roll-out in one Tanzanian district, found that in facilities with ACT in stock, 20% of patients testing positive did not receive ACT, similar to results we present here (Masanja *et al.* 2012).

While the proportion of patients who obtained an ACT significantly decreased from 2010 to 2012, the proportion of patients receiving antibiotics significantly increased to just under half of all patients. An increase in antibiotic prescription after mRDT introduction was also reported by two other studies in mainland Tanzania (Bastiaens *et al.* 2011; D'Acremont *et al.* 2011) and one in Zanzibar (Msellem *et al.* 2009). No specific guidelines were given to health workers on when to prescribe antibiotics as part of mRDT training, but this increase might be expected, given that health workers may be more likely to consider a bacterial diagnosis following negative malaria test results. However, we also observed a significant increase from 31.3% to 47.9% in antibiotic provision among patients not tested between 2010 and 2012. The reason for this is unclear, as antibiotic availability was similarly high during both surveys, and data were not collected on symptoms warranting antibiotic prescription. While data on the aetiology of non-malarial fevers are still limited, a study of children with non-severe febrile illness in northern Tanzania found that while a bacterial pathogen was identified from blood culture in only 0.9% of children, the WHO criteria for pneumonia were met in 48% (Mtove *et al.* 2011). The latter is considered an indication for antibiotics although pneumonia may also be caused by a virus. In Pakistan, a trial assessed withholding of antibiotics among children with fast breathing and did not find a difference in clinical outcomes, suggesting that bacterial pneumonia may be over-diagnosed (Hazir *et al.* 2011). To avoid substituting overtreatment with antimalarials with overtreatment with antibiotics, there is an urgent need for better diagnostics of non-malarial fevers and improved training on case management when patients test negative or when diagnostic tests are not available.

This study has several limitations. The sampling probability of health facilities was based on the most recent malaria outpatient data available, which may have suffered from some inaccuracies. Health workers may have been more likely to perform diagnostic tests and less likely to dispense antimalarials to negative patients as a result of the team's presence. To reduce this possible source of bias, the study team emphasised to health workers the importance of following their normal procedures. Community members may also have been drawn to the facility because of the presence of the study team in hopes of obtaining drugs for both sick household members and as a reserve for future periods of drug stock-outs. Research assistants screened all patients arriving at the facility carefully, but it is possible that some patients may not have had a true illness.

The 2012 survey took place slightly earlier in the year than the 2010 survey. The peaks in malaria incidence in Tanzania usually occur just after the short rainy season in November–December and just after the long rainy season in March–May, which may have meant that in 2010, Mtwara was visited after the peak, and in 2012, Mwanza was visited before it fully developed. However, taking into account variable weather patterns, late rains in 2010, and lack of entomological data, it is not clear that this would have affected parasitaemia prevalence.

Finally, care should be taken in interpreting facility-level data in isolation, as improvements seen at government health facilities may not necessarily translate to an increase in coverage at the community level. IMPACT2 household surveys in the same three regions in 2010 and 2012 similarly showed that among patients visiting public health facilities for fever, there was a significant increase in the proportion receiving a diagnostic test (from 28.7% to 46.6%), and a significant decrease in the proportion obtaining ACTs (from 57.4% to 46.1%) (Thomson *et al*., in draft). However, the household surveys showed no significant change overall in the proportion of patients reporting fever who recalled having a diagnostic test pre- and post-mRDT roll-out (Thomson *et al*., in draft). This appears to have reflected a reduction in the use of public facilities from 25.3% to 16.8% of reported fevers; as diagnostic coverage was much lower in the private sector, no overall increase in parasitological confirmation at the community level was observed despite the mRDT roll-out in the public sector.

Tanzania has made major strides in scaling up access to diagnostic testing for malaria within public health facilities and reducing overuse of ACTs by patients without parasitaemia. However, this study has also demonstrated the dramatically negative role that ACT and mRDT stock-outs continue to play in malaria case management. Several initiatives are underway in Tanzania to improve public sector commodity supply, including text-based stock-out reporting systems (Barrington *et al.* 2010), strengthening of zonal distribution capacity and direct delivery to health facilities, but this continues to be a major challenge. Maintaining buffer supplies of drugs and preventing leakage to the private sector may also potentially reduce stock-outs, as well as ensuring that mRDTs are in stock and appropriately utilised.

Priorities for further research include investigating reasons for under-prescription of ACTs for patients testing positive, and high levels of antibiotic prescription, and the potential effect on antibiotic resistance. As countries increasingly implement policies of universal testing prior to treatment for malaria, it will be important to address these concerns in order to improve case management for febrile illnesses and appropriately target ACTs.
